# A Population Where Men Live As Long As Women: Villagrande Strisaili, Sardinia

**DOI:** 10.4061/2011/153756

**Published:** 2011-10-25

**Authors:** Michel Poulain, Gianni Pes, Luisa Salaris

**Affiliations:** ^1^FNRS, IACCHOS, Université Catholique de Louvain, 6000 Charleroi, Belgium; ^2^Institute for Population Studies, Tallinn University, 10504 Tallinn, Estonia; ^3^Dipartimento di Scienze Biomediche, Università degli Studi di Sassari, 07100 Sassari, Italy; ^4^Dipartimento di Ricerche Economiche e Sociali, Università degli Studi di Cagliari, 09123 Cagliari, Italy

## Abstract

Usually women live longer than men and female centenarians largely outnumber male centenarians. The findings of previous studies identifying a population with a femininity ratio close to 1.0 among centenarians in the mountainous region of Sardinia was the starting point of an in-depth investigation in order to compare mortality trajectories between men and women in that population. The exceptional survival of men compared to women emerges from the comparison with similar Italian data. Age exaggeration for men has been strictly excluded as a result of the age validation procedure. The discussion suggests that besides biological/genetic factors, the behavioral factors including life style, demographic behavior, family support, and community characteristics may play an important role. No single explanation is likely to account for such an exceptional situation and a fully integrated multidisciplinary approach is urgently needed.

## 1. Introduction

In developed countries, it has been widely documented that females live longer than males [1–3]. This female advantage in survival, the so-called Longevity Gender Gap (LGG), results from lower female death rates throughout the lifespan [4–6]. In traditional societies, the LGG was less pronounced and even inversed [7] and a secular trend of increasing LGG is generally observed. Nevertheless the trend has changed in the recent decades resulting in a slightly decreasing LGG [8].

In general, it has been observed that during the life course, the occurrence of certain diseases such as cardiovascular diseases, lung cancer and accidents are more frequent among males [9] and this trend is confirmed when extending the comparison to the top 12 causes of deaths [10]. The cumulative effect of these differences throughout the life course, results in a greater proportion of females surviving at more advanced ages. In populations experiencing a low mortality, the femininity ratio (F/M) among centenarians is usually above 4, that is, there are more than 4 female centenarians per male centenarian (data source: *Human Mortality Database*). However, recent research has shown that in certain populations the F/M ratio among the oldest olds may be remarkably lower, and when this occurs it is often the result of a reduced excess of male mortality rather than a higher mortality of women [11–13]. Among these populations, the one living in the Mediterranean island of Sardinia certainly represents an interesting case study. This region has been suspected to be characterized by a particularly high male longevity [14]. Considering the importance of age validation in longevity studies, the demographic data were carefully checked by using all available data sources in order to avoid cases of age exaggeration [15]. Based on these validation results, an area with significantly higher levels of longevity and lower F/M ratio among centenarians has been identified in the central-eastern part of the island and was called the *Blue Zone* [16]. As a result the topic of research has been switched from individual longevity to population longevity aiming to facilitate the identification of longevity determinants shared by people living in such area.

This longevity *Blue Zone* located in the provinces of Ogliastra and Nuoro in the mountainous region of Sardinia shows a value of the Extreme Longevity Index (ELI^1^) computed for the newborns between 1880 and 1900 that is more than twice as high as that of whole Sardinia. Strikingly the F/M ratio among centenarians in this population is close to one, as 47 male centenarians were found and only 44 female centenarians. Thus the LGG usually observed among the oldest olds in other populations is virtually nonexistent in the longevity *Blue Zone*.

From these findings, the following question arises: *How is it possible that in this population the proportion of men reaching the extreme end of age spectrum is the same as for women? *In order to answer this question, the population of the municipality of Villagrande Strisaili was selected, as it is the municipality with the highest ELI value in Sardinia (10.8 centenarians per 1,000 newborns) and an extraordinary low F/M ratio; among the 30 centenarians found in Villagrande 16 are male. The life trajectory of each individual born in the village during the period 1876–1912^2^ was followed from birth to death. 

The analysis of the collected data allows the comparison of the mortality trajectory for men and women. Moreover the life table estimates of Villagrande have been compared with the corresponding ones for Italy extracted from the *Human Mortality Database *(HMD) [17]. The analysis of mortality trajectories allows the identification in more detail of the critical age interval for determining between-gender level differentials in longevity among the populations considered.

In the discussion, possible explanations for the lack of LGG observed in the *Blue Zone* will be reviewed in light of existing literature. As we will suggest, besides biological/genetic factors, numerous behavioral characteristics may count for explaining the male advantage. Accordingly only multidisciplinary investigations could explain the exceptional survival of males in the population under study. Having in mind this objective the few empirical evidences presented here should be considered as a starting point for further investigations.

## 2. Setting, Data, and Methods

### 2.1. Setting

The village of Villagrande is located at 700 meters above sea level in the province of Ogliastra, but the altitude of its territory ranges from sea level to *Punta La Marmora *at 1,834 meters. On 1 January 2010, 3,441 inhabitants lived in Villagrande (ISTAT) where agropastoralpastoral activities and traditional life style are still prevalent. Despite the fact that until the 1960s this region was among the poorest within the island, recent economic developments brought the population of this area close to the average welfare standard of the Italian population.

### 2.2. Data

The database developed for the present study includes all individuals born in Villagrande from 1876 to 1912. For each individual we traced the exact date at death or the proof that he/she was still alive at the date of investigation. The data was gathered from civil registers (which record all births, marriages, and deaths), parish registers and the population register (*anagrafe*). All information has been collected in the municipality population registration office and was cross-checked with information reported in the military register and orally reported information from any relatives of these persons. With regard to those who died outside the village, the information was recovered by using annotations on date and place of death reported in the margin of the birth certificate or transcription of the date of death in the *anagrafe*. For those who emigrated and for whom no death has been reported, the survival status has been verified with the municipality of current residence.

A total number of 1,957 persons born in Villagrande during the years 1876 to 1912^3^ have been considered in this study of which:

- 1,624 who died in Villagrande itself;
- 206 who died outside Villagrande;
- 20 who were still alive at the date of investigation;^4^
- 107 for whom the date of death or the survival is unknown.

### 2.3. Coverage

The data collection method enables to reduce significantly the number of missing dates of death due to lack of information (107 cases). The latter group of newborns has been divided between outmigrants with the documented date of emigration and those where no indication of emigration was available. For those without information on emigration, their date of marriage or the latest trace in administrative documents, for example, military reports was considered as partial information. As a result, there were only 26 newborns for whom no information at all on survival was found, whereas for other 81 we found at least information attesting their survival at the time of marriage, military examination, or emigration. A final coverage rate of above 98 per cent was reached. This should be considered exceptional in the context of historical demography and family reconstruction. Moreover such level of coverage contributed to the complete validation of the database and the strength of the subsequent analysis.

### 2.4. Methods

The presence of still alive newborns and newborns with partial information implied the setting up of censoring strategies in order not to lose precious information. The general rule was to take the last information available that attested survival as censoring time.

The method utilized in this study is the classical cohort life table construction privileging as output the mortality rates and the cumulative survival curve. The computation of the survival curve takes into account the contribution of all censored newborns up to their exit of observation. The exclusion of the 26 persons without information and the censoring of the 101 others (20 alive and 81 with partial information) can hardly affect the validity of our findings. The distribution of the 1957 persons by gender, five years age groups at death or survival is displayed in Table 1.

For comparative purposes, we utilized data for Italy collected from birth cohort life tables available in the HMD for the same birth cohorts 1876–1912 considered for Villagrande and involving separately males and females by single year of birth and age.

## 3. Results

**Figure 1:**
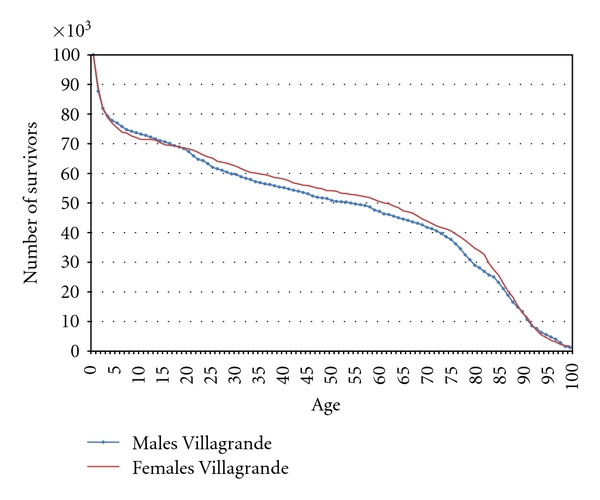
Comparing survival curves for males and females in Villagrande for a hypothetical cohort of 100,000 newborns of the years 1876–1912.

The survival curves from birth till age 100 are shown in Figure 1 separately for men and women by considering all birth cohorts 1876–1912 together. The corresponding data are reported in Table 2.

At age 5, one newborn out of four has already died and an additional tenth did not reach the age of 18. At age 50, 46.4% of males and 53.3% of females are surviving. From age 50 to 75, males and females seem to be exposed to the same mortality risk. Between age 75 and 90, men record a higher level of survival compared to females so that finally at age 90 about 12% of the newborns—both for males and females—are surviving. 

**Figure 2:**
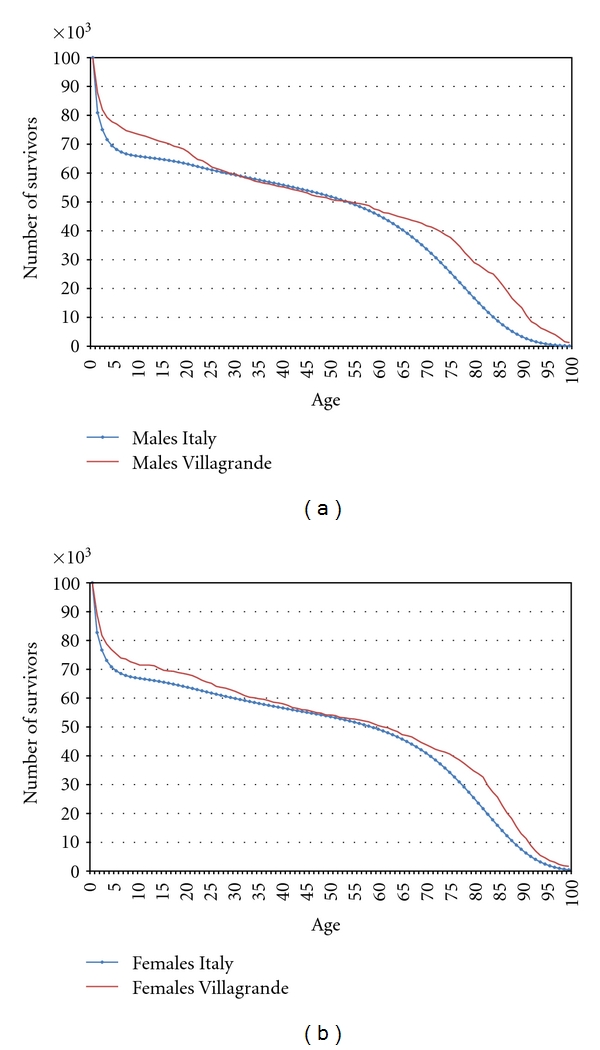
(a) Comparison of survival curves for males in Villagrande with the corresponding data extracted from the Human Mortality Database for the whole of Italy (birth cohorts from 1876 till 1912 included). (b) Comparing survival for females in Villagrande with strictly comparable data extracted from the Human Mortality Database for the whole of Italy (birth cohorts from 1876 till 1912 included).

The comparison with strictly comparable data for Italy extracted from the Human Mortality Database is presented in Figures 2(a) and 2(b) separately for men and women. Compared to the Italian population the situation is really exceptional in Villagrande. For infant and child mortality two major demographic features emerge; in Villagrande a lower mortality up to 2 years old than in Italy was recorded, while from age 2 to 5 years old mortality estimates increase. This particular trend was observed by Coletti for Sardinia already in 1908 [18]. This low trend in early mortality could not be explained in terms of major economic development, given the poor conditions of Sardinian population at the end of 19th century, but the author hypothesizes that rather it was determined by the positive effect of the common practice of prolonged breastfeeding and the limited participation of Sardinian women to external works. Subsequent studies on regional data [19, 20] and on selected villages confirmed this trend of low early mortality [21, 22].

Considering the mortality rates from age 5 until age 18, it emerges that once a newborn managed to reach 5 years old, he/she faced more favourable conditions for survival. Nevertheless we observe in Villagrande a higher risk of death from age 6 to 10 compared to the mainland, while estimates until 18 are similar in the two populations. Therefore the survival advantage at early ages of individuals born in Villagrande Strisaili is not maintained, but generates overmortality at subsequent ages. This changing trend might be interpreted in terms of different timing in the selection process of frail individuals. In the Italian population frails are eliminated in the first years of life, while in Villagrande Strisaili this process appears to be postponed.

The analysis of adult mortality appears to be more complex since the latter is mainly due to external causes such as losses in WW I for men and maternal mortality for women. Military data show that no less than 40 males born in Villagrande died during WW I and that most of them were born in the period 1890–1900. For women, maternal mortality was also high during the reproduction period. Orrù and Putzolu [23], in their study on the diffusion of professional assistants for delivery, pointed out that in 1899 there was no professional assistant in Villagrande and in the surrounding area. However, in the village, a maternal mortality rate varying between 10.6 and 11.1 per 1,000 births was estimated [24], which is in line with the levels recorded in other Sardinian villages and in other European countries for the same period [25]. Due to the higher mortality risk for both sexes between 18 and 50, the proportions of 46.4% of men and 53.3% of women surviving at age 50 are similar to the ones observed for the whole of Italy (Figures 2(a) and 2(b)).

**Figure 3:**
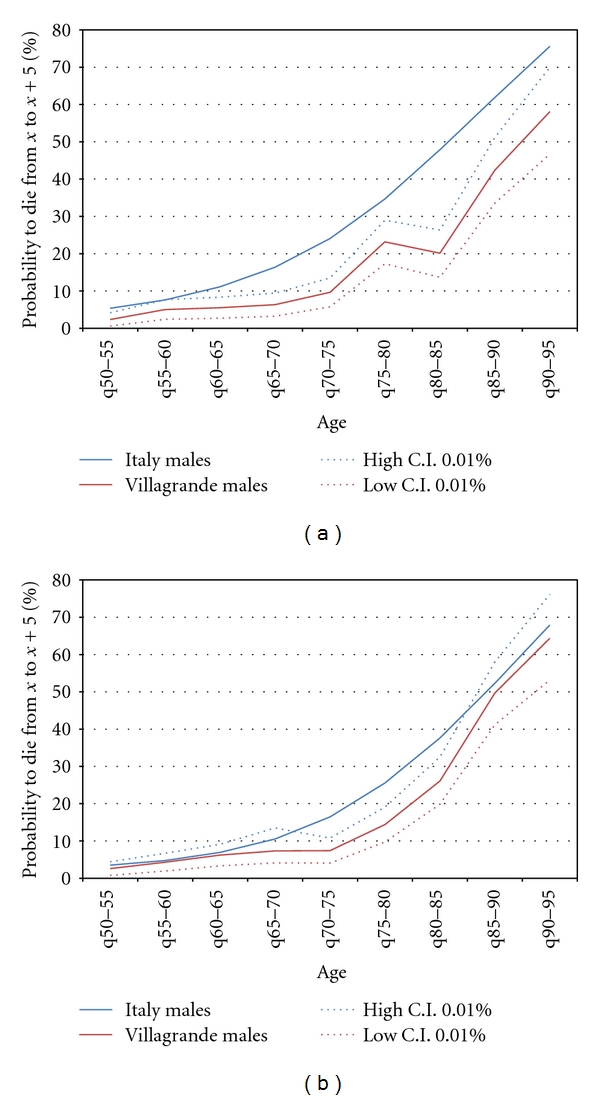
(a) Compared five years' probability of dying for males in Villagrande with strictly comparable data extracted from the HMD for the whole Italy (birth cohorts from 1876 till 1912 included). (b) Compared five years' probability of dying for females in Villagrande with strictly comparable data extracted from the HMD for the whole Italy (birth cohorts from 1876 till 1912 included).

About three out of four males and females surviving at age 50 finally reached age 75. When comparing these figures with the ones of the Italian population a lower mortality risk is evident mostly for men. (For men, 74% of those aged 50 survive up to 75 for Villagrande compared to 49% in Italy. For women these proportions are, respectively, 75% and 64%.) The lower level of the mortality risk for men is confirmed when comparing the mortality rates (Figures 3(a) and 3(b)). For men the mortality rates are significantly lower starting at age 60 (considering a confidence interval of 0.01) while for women mortality rates are also lower but significant only from 70 to 85 and no longer significant after that age. Finally, it is worth noting that the proportion of those surviving at 90 years in the life table computed for Villagrande is balanced almost evenly between men and women (10,7% for men and 11,4% for women), and this result must be considered exceptional compared with the situation in Italy.

## 4. Discussion

Why do men usually live shorter than women? This question has been addressed in several studies where potential explanatory factors are examined [10, 26–33]. Why men live as long as women in Villagrande and, more generally, in the Sardinian longevity *Blue Zone,* is a related question emerging from the results of this study. To discuss this question more in detail, we will review several factors proposed in the literature to explain the LGG advantaging females and try to understand why they seem not to work in the population under concern. In addition some other factors that may directly favor male longevity will be considered.

### 4.1. Biological/Genetic Factors

The first group of factors invoked to explain the female advantage in longevity relates to biological traits. An increased level of homozygosity in the population, which may reduce the variability of the genetic pool, has been reported to favor male longevity [34]. As almost everywhere in the whole *Blue Zone*, the population of Villagrande was characterized by a high level of endogamy until WW II [35, 36]. In addition, the role of gender-specific genes in longevity has been largely investigated. Women have two X chromosomes, one from their father and the other from their mother, while males have only one X chromosome and one Y chromosome. Because of this, women have two cell lines and if any recessive allele detrimental for survival is inherited, the allele on the second X chromosome could compensate the expression of the first one, a protective strategy that is lacking in males [37]. On the other hand, an increased mutation load in the X chromosome may result from the higher paternal age at reproduction, a condition relatively common in central Sardinia, and that may contribute to reduce life span only in daughters [38], as sons do not inherit the paternal X chromosome. One possibility is that in genetically homogeneous populations such as the one living in Central Sardinia [39], the probability that females carry the same alleles in both X chromosomes (homozygosity) is so high as to reduce the protective effect of having two X chromosomes. Alternatively, specific X-linked recessive alleles may exist in the *Blue Zone *population, as a result of local selective pressures, that may extend the life expectancy of men via different mechanisms. This may be the possible effect of G6PD deficiency, an X-linked disorder quite common in the island [40] that was hypothesized to explain Sardinian longevity for males [41]. Unfortunately the few available epidemiological data [42] do not support the existence of a higher prevalence of G6PD deficiency in villages with increased longevity. Recent findings suggest that an X-linked component affects the telomere length and that brings an additional potential explanation for the LGG as the telomere length is highly inherited, longer in women and linked to survival [43, 44]. 

It has also been hypothesized that Y-chromosome allelic variants may affect male survival, probably influencing the circulating levels of testosterone and hence its biological effects on vasculature and other tissues. Men with higher testosterone levels show reduced markers of inflammation and metabolic syndrome and decreased risk of mortality for all causes independently from the overall health status [45]. Although the study of the endocrine profile of Sardinian oldest olds showed markedly higher testosterone serum levels and lower estradiol than in younger controls [46] the real significance of these results is still to be clarified. Moreover, a study carried out in a limited number of Sardinian male centenarians and younger control subjects failed to find any significant association between longevity and Y-related markers [47], although an additional set of markers and a larger sample may be required to further clarify the role of Y chromosome on increased survival.

The role of mitochondrial DNA on gender differential longevity has also been investigated. In particular, the frequency of a polymorphic variant called J haplogroup was reported to be increased in male centenarians from continental Italy [48]. In the Ogliastra population, the frequency of such haplogroup in the whole population is more than twice the average frequency in Sardinia (18.3% versus 6.7%) [49], making this polymorphism a valuable candidate to explain the reduced excess male mortality.

Other genetic traits have been claimed to favor male longevity, such as the *β*-thalassemia trait that confers some “protection” against the development of premature cardiovascular diseases [50]. Caselli and Lipsi [51] showed that the population in the *Blue Zone* presents the lowest mortality in Sardinia but also the lowest cardiovascular mortality. They concluded that these findings indicate the existence of a specific genetic or environmental factor that protects Sardinian men, further research being needed to clarify the exact nature of these factors.

Potential explanatory factors in this first group also include anthropometric differences often observed between males and females. The difference in average height between male and female may be lower in the *Blue Zone* population, while the average body mass index of females may be relatively higher compared to males. If these two hypotheses are confirmed this would lead to a longevity advantage for males as some studies have shown that there is an inverse relationship between both height and body mass index and longevity [52, 53]. Evidence derived from military conscripts shows a shorter height on average for men in the *Blue Zone* compared to those in the whole of Sardinia [54], whereas no such data are available for women, thus precluding the possibility to test this hypothesis.

### 4.2. Behavioral and Sociocultural Factors

The second group of factors is related to individual behavior and Sociocultural traits of the population. First of all it is important to mention that migration does not introduce any bias in our results as all and only individuals born in Villagrande are included in the study even though some of them later emigrated outside the village and did not die in the village. By evidence these persons who emigrated from the village could be different with regard to behavior and/or social characteristics from those who stay living in the village all their life. Nevertheless it has a limited impact as emigrants represent less than 10% of the newborns.

 Other individual behaviors include possible differences in nutrition between males and females, but also in physical activity and energy balance. Such differences in behavior are mostly linked to the different occupations of males and females as well as other aspects of their life style. Although no specific study up to now ascertained any gender-related differences in Sardinian nutrition, it may be hypothesized that the distinct role of women within the families and the prevalent occupation of men may have induced appreciable differences in dietary patterns between genders. Also hypothetical difference in feeding boys and girls in their childhood should be investigated. Drinking red wine from Ogliastra was claimed to have a positive effect on longevity due to its higher content of antioxidants [55]. Combined with the traditional Sardinian higher consumption of wine by males compared to females, this may hypothetically contribute to male longevity. Besides, as in the longevity *Blue Zone* men were mostly shepherds while women were mostly responsible for all domestic tasks including animal breeding and vegetable and cereal cultivation [56], men likely had different physical activities compared to women during their active life that lasted until advanced ages. The additional circumstance of living in a mountainous area increases the daily energy expenditure of most men compared to women, also favoring longevity [57, 58]. Women were the main responsible for the care of goods and property but also the trustees of transmission of the ethical values, including self-defense, implying a certain degree of aggressivity [59]. Some specific character traits of males in the *Blue Zone* compared to females like a better tolerance to stress, a high level of extraversion and sociability, and possibly a better ability to take advantage of the positive aspects of the health transition, may also play a positive role in their longevity especially by lowering cardiovascular mortality [60]. However, data on personality characteristics of men in Villagrande is currently not available and only future research will allow testing this hypothesis.

Accordingly, the absence of a gender gap in mortality risk might also be attributed to differences in men's and women's social behavior within a rather archaic society that acknowledges distinct roles to males and females. In particular, the population of central Sardinia during the centuries experienced a kind of matriarchal organization [61] that lasted till the onset of industrialization in the late 1950s. Nevertheless the concept of a “matriarchal organization” in Sardinia, as it was put by Pitzalis-Acciaro in 1978 [61], is a controversial one and has been challenged by anthropologists thereafter [62, 63]. Anyway, during the last few centuries Sardinian society was clearly dominated by men, as elsewhere in the Mediterranean countries, especially among rural communities of farmers. The situation may have been slightly different among pastoral communities, where the chronic absence of men increased the workload and responsibility of women. This does not mean, however, that women had a real power; instead they had to shoulder the toughest jobs and accordingly it could be better to refer to a “women-centered” society, rather than “matriarchal” [64]. This specific role of women within the family, and hence within the community, probably implied an increased “chronic stress burden” compared to men, which ultimately may have contributed to disfavor female longevity [63].

Some specific Sociocultural traits of the population living in the *Blue Zone* may also favor male longevity. Some gender stereotypes and related social norms may favor men compared to women and this seems to be the case in Ogliastra [59] where oldest men are the target of intense attention within the family but also in the local community, which may bring them support for living longer. Also it is well established that a stronger family support and long-standing living arrangement in a married couple may help to live longer [65]. If men marry younger women, they will largely escape widowhood that has a negative impact for living longer at least for men. Moreover, a high level of remarriage among widowed men increases the age difference with their last spouse and keeps a high proportion of men still married in old ages. Among the 324 ever-married men of Villagrande who died above age 80, 41 (13%) have been married more than once and the age difference with their (last) spouse, for 136 of them (39%), was larger than 10 years (median 9.1) while only 25 (7%) had an older spouse. Such larger age differences between spouses are favorable for male longevity [66]. In addition, after widowhood, older women tend to live more often alone while older men will live preferably with an unmarried child taking into account that entering a nursing home is an exception for the population concerned. Overall this situation also favors male longevity.

Furthermore the possible role of fertility and more specifically the impact of the timing of the reproductive period on women's survival have to be considered. Historically, Sardinian women were characterized by high fertility and an older age at birth of their children [67]. Astolfi et al. [68] shows that the spatial pattern of late reproduction behavior in Sardinia display areas that are very similar to the longevity *Blue Zone* assuming that there exists some common explanation linking late reproduction behavior and longevity. Such association has been proved in other populations [69, 70] but only as far as female longevity is concerned. Accordingly such argument cannot be considered as improving longevity of males compared to females. Nevertheless a more recent investigation on large historical databases [71] shows that late reproductive behavior is not only associated with female postmenopausal longevity but also with survival past age 50 of brothers of late-fertile women. According to these authors, these results support the hypothesis that both the late female fertility and the slow somatic aging for both men and women may be promoted by the same genetic variants. These arguments could explain the relative higher longevity observed in the *Blue Zone* but not the male advantage identified in the population under study. Nevertheless the similarities observed between the spatial patterns of late fertility and high longevity could be related to higher endogamy.

## 5. Conclusion

An intensive data collection and complete family reconstruction allowed finding the date of death or confirming the survival at the date of survey of almost all 1957 children born in Villagrande during the years 1876–1912. The cohort life table has been estimated and the mortality trajectory identified separately for men and women. The survival curves and mortality rates have been compared with similar ones for Italy extracted from the HMD. The exceptional situation of men in Villagrande emerges from that comparison, as men are living as long as women do, which is unusual elsewhere. Three conclusions can be drawn from our preliminary investigations. First age exaggeration for men has been clearly excluded as a result of the age validation exercise among the possible reasons explaining why men apparently live as long as women in Villagrande, as well as in the Sardinian *Blue Zone*. Secondly, we consider that the above-mentioned factors may work jointly in a population characterized by a sort of “matriarchal” organization but the overall impact of these factors should be strong enough to explain why men are living as long as women do in that population, a significantly different situation compared to all populations in developed countries.

Thirdly, it is unlikely that a single explanation can be found for justifying such an exceptional situation. Quoting Franceschi et al. [11] “*besides the classical biomedical disciplines such as gerontology, geriatrics, genetics and immunology among others, a new integrated approach, including demography, historical demography, anthropology, and other social sciences, appears to be necessary to disentangle the complex interaction between the environmental/cultural and biological/genetic components responsible for sex differences in centenarians.*”

The results of this investigation indicate that the usually observed higher mortality for men is not valid for the population of Villagrande and, by extension, to the one of the *Blue Zone*. Among the whole list of potential factors only few evidences have been found so far. The explanatory research should be continued keeping in mind that only a multidisciplinary approach will help finding the underlying set of explanatory factors.

## Figures and Tables

**Table 1:** Observed number of deaths (showing also censored cases) by gender and age groups and cohorts of newborns including those who were alive at the time of investigation (2006).

	Males					Females				
Age completed	1876–1889	1890–1900	1901–1912	Alive	Total	1876–1889	1890–1900	1901–1912	Alive	Total

0	27 (1)	37 (1)	58 (1)	—	125	38 (1)	31 (1)	34 (1)	—	106
1–4	36 (2)	41	29 (1)	—	109	48	42	35	—	125
5–9	9	11	17	—	37	21	7	9	—	37
10–14	7	7	12	—	26	4	2	11	—	17
15–19	6	14 (6)	13 (3)	—	42	4	5	4 (2)	—	15
20–24	6 (1)	32 (5)	14 (2)	—	60	6	17	6	—	29
25–29	7 (5)	9 (1)	8 (1)	—	31	9 (2)	9	8	—	28
30–34	12 (7)	5 (4)	9 (3)	—	40	8 (1)	9	7 (2)	—	27
35–39	7 (2)	1 (1)	7 (3)	—	21	10 (1)	2	4	—	17
40–44	4 (1)	4	11 (2)	—	22	10	5	5	—	20
45–49	3	9	8 (1)	—	21	7	4	5	—	16
50–54	7	2	3	—	12	3 (1)	6	4	—	14
55–59	13	5	5	—	23	5	5	11	—	21
60–64	10	6	6 (1)	—	23	13	5	11	—	29
65–69	14	2	8	—	24	9	13	11	—	33
70–74	9	12	15 (2)	—	38	11	9	9	—	29
75–79	24	25	30 (1)	—	80	16	18	18	—	52
80–84	9	13	29 (2)	—	53	29	25	28 (4)	—	86
85–89	30 (1)	22	35	—	88	26	38	52 (2)	—	118
90–94	25	15	29 (2)	2	73	20	17	37	4	78
95–99	12	10	11	6	39	7	6	9	5	27
100+	3	3	2	1	9	7	1	1	2	11
Total	** 280 (20)**	** 285 (18)**	**359 (25)**	**9**	996	**311 (6)**	**276 (1)**	**319 (11)**	**11**	935
Unknown	**3**	**3**	**5**	—	11	**6**	**3**	**6**	—	15

**Table 2:** Comparison of survival curves for males and females in Villagrande with the corresponding data extracted from the *Human Mortality Database* for the whole Italy (birth cohorts from 1876 till 1912 included).

Age	Villagrande		Italy	
	Males	Females	Males	Females

0	100000	100000	100000	100000
5	76506	75294	68333	69639
10	72791	71337	65950	67028
15	70181	69519	64851	65706
20	65964	67914	62907	63940
25	59940	64813	59438	61947
30	56827	61818	56845	60087
35	52811	58930	54591	58368
40	50703	57112	52648	56795
45	48494	54973	50797	55320
50	46386	53262	48773	53801
55	45181	51765	46176	51927
60	42871	49519	42676	49483
65	40562	46417	37929	46079
70	38153	42888	31743	41275
75	34337	39786	24114	34525
80	26305	34225	15779	25754
85	20984	25027	8258	16098
90	12149	12406	3162	7694
95	4819	4064	777	2480
100	904	1176	105	456
Probability to survive from 50 to 75				
	74,0%	74,7%	49,4%	64,2%

## Endnotes

1. ELI (extreme longevity index) is the proportion of newborns in a given place who became centenarian wherever and thus estimates the probability for a newborn in a given place to reach the age 100. In the *Blue Zone*, 91 centenarians were found among the 17,865 newborns of the years 1880–1900 while 1132 centenarians were counted among the 516,276 newborns in the whole Sardinia. The ELI values are, respectively, 5.1 centenarians per 1,000 newborns in the *Blue Zone* compared to 2.1 for Sardinia.
2. Although the data are available since 1866, the year when the civil registers were first held in Sardinian municipalities, only the cohorts born after 1876 are considered, as the data related to the earlier birth cohorts are less complete. The last year of observation, 1912 has been chosen in order to include the cohorts of those who were at least 95 years old at the time of investigation and to exclude those whose infant mortality could be increased during WW I.
3. From the totality of those born in Villagrande were excluded only those babies (120 out of 2077) that clearly were accidentally born in the village, where their parents were living at that time for work-related reasons. To identify these newborns we utilized the origins of both parents and their occupation (miners, workers involved in the construction of roads and train networks, police officers) as usually reported in the birth certificate.
4. As of 5 September 2006.
